# Reference data set for a Norwegian medium voltage power distribution system

**DOI:** 10.1016/j.dib.2023.109025

**Published:** 2023-03-01

**Authors:** Iver Bakken Sperstad, Olav Bjarte Fosso, Sigurd Hofsmo Jakobsen, Arnt Ove Eggen, Julie Helen Evenstuen, Gerd Kjølle

**Affiliations:** a SINTEF Energy Research, Trondheim, Norway; b NTNU – Norwegian University of Science and Technology, Trondheim, Norway

**Keywords:** Grid data, Load data, Test grid, Test network, Test system, Benchmark system

## Abstract

This article describes a reference data set for a representative Norwegian radial, medium voltage (MV) electric power distribution system operated at 22 kV. The data set is developed in the Norwegian research centre CINELDI and will in brief be referred to as the CINELDI MV reference system. Data for a real Norwegian distribution system were provided by a distribution grid company. The data have been anonymized and processed to obtain a simplified but still realistic grid model with 124 nodes. The first part of the data set describes the base version of the reference system that represents the present-day state of the grid, including information on topology, electrical parameters, and existing load points. The data set also comprises a load data set with load demand time series for a year with hourly resolution and scenarios for the possible long-term load development. These data describe an extended version of the reference system with information on possible new load points being added to the system in the future. A third part of the data set is data necessary for carrying out reliability of supply analyses for the system. The base version of the reference system described in this article can be extended to represent other types of distribution grids (e.g., with a ring topology). The reference grid can be used for assessing new methods and principles for distribution system operation and planning, including assessment of flexibility resources, active distribution grid measures, grid reinforcement planning, grid reinvestment planning, reliability of supply analysis, self-healing, etc.

**Specifications Table**

**Table:** 

Subject	Electrical and Electronic Engineering
Specific subject area	Electrical power distribution systems
Type of data	Tables
How the data were acquired	Grid data were provided by a Norwegian distribution grid company for a medium voltage (22 kV) distribution system. These data were acquired from the grid company's Network Information System (NIS).
Data format	Secondary
Description of data collection	Raw data acquired for a real power distribution system had to be anonymized due to confidentiality and were then processed to obtain a simplified, representative reference data set for a Norwegian distribution system. The data processing and anonymization procedure is described in detail in the *Experimental Design, Materials, and Methods* section.
Data source location	Country: Norway To preserve anonymity, no further details can be specified about the location of the real distribution system from which data were collected*.* In addition, the Norwegian handbook for grid planning [1] is source of supplementary technical and economic data on distribution grid components.
Data accessibility	Repository name: Reference data set for a Norwegian medium voltage power distribution system DOI:10.5281/zenodo.7703070 (The raw data that the reference data set is based on are confidential.)

## Value of the Data

• This reference data set was initially created for use in the Norwegian research centre CINELDI^1^ to benefit partners from all parts of the Norwegian power distribution industry, including distribution grid companies, research providers, technology providers and innovators, public authorities, and member organizations. When doing research on challenges and conditions specific to a given country, such as Norway, it has been useful to establish a reference system representative for a Norwegian distribution system. For this purpose none of the existing international test grids, reference grids or benchmark systems that are in use in the scientific community [2], [3], [4], [5], [6] were suitable.
• The base version of the reference system represents present-day conditions, but the data set also includes extensions for representing upcoming challenges with new loads (e.g., fast charging stations). Being able to study such challenges and how to solve them is relevant to stakeholders in Norway and potentially also in other countries.
• In addition to the value of the data specific to Norwegian stakeholders, it also has added value for international use compared to existing reference grids since it includes information such as: line length, component age, investment costs^2^, load demand time series for a full year, long-term load development scenarios, and necessary data for reliability of supply analysis.
• The data set includes a summary of technical and economic data for the most used MV distribution grid components in Norway that will be valuable also for creating data sets for other MV distribution grids than the reference grid presented here.
• The base version of the reference grid presented here will be used as the basis for extensions for reliability of supply studies (e.g., grids with ring connections, or several interconnected distribution systems). It is also a relevant basis for extensions necessary for studies of cyber-physical power systems (including reference data for ICT infrastructure as well as power grid infrastructure).
• The reference system can be used for assessing new methods and principles for distribution system operation and planning, such as grid reinforcement planning, reinvestment planning, reliability of supply analysis, self-healing, etc. Including load time series makes it possible to simulate system operation utilizing flexibility resources and to account for active measures in distribution grid planning studies. The data set will in the future be complemented by a separate data set for load demand of fast-charging stations for electric vehicles, which is a relevant example of a flexibility resource.
• The MV reference system represents distribution substations and the underlying low voltage (LV) distribution grids as aggregated load points, but it can be combined with existing and future data sets for LV grids. Moreover, although the data set contains a very simple representation of local energy communities, it can also be combined with detailed data on consumers and prosumers connected to LV grids, such as a recent data set for Norwegian energy communities [7]. The MV reference system can also be combined with existing and future data sets for the upstream high voltage (HV) regional distribution grid (sometimes also referred to as a sub-transmission grid).

## 1 Data Description

This article presents a representative reference data set for a Norwegian medium voltage electric power distribution system. The data describe both a base version of the reference system, which represents the present-day state of the grid, and extensions. It is a combined data set comprising three parts:

(i) A grid data set that describes the base reference system. It comprises information on topology, electrical parameters, and existing load points with peak load values.
(ii) A load data set with relative load demand time series for a year with hourly resolution and scenarios for the possible long-term development of peak load. These data describe an extended version of the reference system with information on possible new load points and flexibility resources being added to the system in the future.
(iii) Necessary data for carrying out reliability of supply analysis for the system.

The three parts of the data set are described in detail in the following three subsections.

## 2 Grid Data

Table 1 gives an overview of the files in the grid data part of the reference data set.

**Table 1:** Overview of the grid data set.

File name	Description of data
CINELDI_MV_reference_grid_base_bus.csv	Bus (node) data on the MATPOWER case format [8], including peak load data for each of the nodes with load points
CINELDI_MV_reference_grid_base_bus_extra.csv	Extra data fields for the load points (at a subset of the nodes), in addition to the bus data defined in the standard MATPOWER case format [8]: ZIP load model parameters
CINELDI_MV_reference_grid_base_branch.csv	Data for the branches (distribution lines) on the MATPOWER case format [8]
CINELDI_MV_reference_grid_base_branch_extra.csv	Extra data fields for the branch data, in addition to those defined in the standard MATPOWER case format [8]: length, type, installation year, and type of location
CINELDI_MV_reference_grid_base_branch_pf_sol.csv	Power flow for the base reference system
CINELDI_MV_reference_grid_base.xls	Excel file with five spreadsheets containing the same data as in the .csv files
standard_underground_cable_types.csv	Technical and economic data for relevant new underground cables in the reference system
standard_overhead_line_types.csv	Technical and economic data for relevant new overhead lines in the reference system
standard_substation_types.csv	Technical and economic data for relevant distribution substations that can be added to the reference system
distribution_line_types_in_reference_grid.csv	Overview of line types present in the original grid data set

**Fig. 1:**
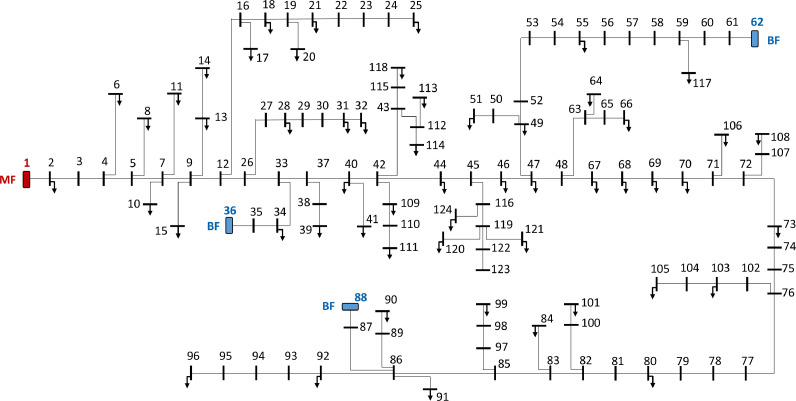
CINELDI MV reference system, with one main feeder (MF) and three backup feeders (BF).

Fig. 1 shows a single-line diagram that gives an overview of the reference system. It is a radial medium voltage (22 kV) distribution system with 124 nodes^3^ and 123 distribution lines^4^. The distribution lines are a mix of underground cables and overhead lines.

The main feeder (MF) is located at node 1, and the three backup feeders (BF) are located at nodes 36, 62 and 88. The main feeder is connected to a high-voltage (HV) distribution grid, sometimes referred to as a sub-transmission or regional distribution grid. The three backup feeders are connected to a neighbouring distribution system at the same voltage level (22 kV). It is assumed that the available power capacities at the main feeder and the backup feeders are sufficient to cover the total peak load of the reference system.^5^ The transformer connecting the MF node of the MV grid to the HV grid is not included in the data set. Neither does the data set include distribution transformers connecting the MV grid to downstream low voltage (LV) grids, and load demand is represented through aggregated load points at a subset of the MV nodes. The main parameters characterizing the base reference system are described in Table 2.

**Table 2:** The main characteristics of the base reference system.

Parameter	Value
Number of nodes	124
Voltage level (base voltage)	22 kV
Base power	10 MVA
Number of load points	54
Total peak load demand, real power	6.407 MW
Total peak load demand, reactive power	2.106 MVAr
Power factor of loads	0.95 (lagging)

### 2.1 Technical data for the reference grid

Table 3 shows an extract of the data file *CINELDI_MV_reference_grid_base_bus.csv* that describes the bus (node) data on the MATPOWER case format [8].

**Table 3:** Example of bus data from CINELDI_MV_reference_grid_base_bus.csv.

bus_i	bus_ type	Pd	Qd	Gs	Bs	bus_ area	Vm	Va	base_kV	zone	Vmax	Vmin
1	3	0	0	0	0	0	1	0	22	0	1.05	0.95
2	1	0.1025	0.0337	0	0	0	0.9985	−0.0733	22	0	1.05	0.95
…	…	…	…	…	…	…	…	…	…	…	…	…
124	1	0.1535	0.0504	0	0	0	0.9670	−0.6645	22	0	1.05	0.95

The data columns in Table 3 are defined as follows (based on [8]):

• bus_i: Bus (node) number (positive integer)
• bus_type: Bus type (1 = PQ, 2 = PV, 3 = ref, 4 = isolated)
• Pd: Real power for peak load demand (MW)
• Qd: Reactive power for peak load demand (MVAr)
• Gs: Shunt conductance (MW demanded at *V* = 1.0 p.u.)
• Bs: Shunt susceptance (MVAr injected at *V* = 1.0 p.u.)
• bus_area: Area number (positive integer)
• Vm: Voltage magnitude (p.u.)
• Va: Voltage angle (degrees)
• base_kV: Base voltage (kV)
• zone: Loss zone (positive integer)
• Vmax: Maximum voltage magnitude (p.u.)
• Vmin: Minimum voltage magnitude (p.u.)

The nodes with positive values in the Pd column are defined as existing load points in the system. The complex voltage values stored in the Vm and Va columns are obtained by power flow calculations given the base reference system data with the load demand values in the Pd and Qd columns. The values in the Vmax and Vmin specify bus voltage limits ±0.05 p.u. for the grid, which are voltage limits that are commonly used as a planning criterion for Norwegian MV distribution grids.

Table 4 shows an extract of the data file *CINELDI_MV_reference_grid_base_branch.csv* that describes the branch (distribution line) data on the MATPOWER case format [8].

**Table 4:** Example of branch (distribution line) data from CINELDI_MV_reference_grid_base_branch.csv.

f_bus	t_bus	br_r	br_x	br_b	rate_A	rate_B	rate_C	tap	shift	br_ status
1	2	0.00166	0.00239	0.00274	17.71888	0	0	0	0	1
2	3	0.00103	0.00149	0.00170	17.71888	0	0	0	0	1
3	4	0.00190	0.00167	0.00156	15.24205	0	0	0	0	1
…	…	…	…	…	..	…	…	…	…	…

The data columns in Table 4 are defined as follows (based on [8]):

• f_bus: “From” bus (node) number
• t_bus: “To” bus (node) number
• br_r: Resistance (p.u.)
• br_x: Reactance (p.u.)
• br_b: Total line charging susceptance (p.u.)
• rate_A: MVA rating A (long term rating), set to 0 for unlimited
• rate_B: MVA rating B (short term rating), set to 0 for unlimited
• rate_C: MVA rating C (emergency term rating), set to 0 for unlimited
• tap: Transformer off nominal turns ratio, (taps at “from” bus, impedance at “to” bus, i.e., if *r* = *x* = *b* = 0, tap = |Vf|/|Vt|, where Vf and Vt are bus voltages at the from and to bus, respectively)
• shift: Transformer phase shift angle (degrees), positive ⇒ delay
• br_status: Initial branch status, 1 = in-service, 0 = out-of-service

The solution for the power flow for the base reference system is stored in the file *CINELDI_MV_reference_grid_base_branch_pf_sol.csv*, with the following columns:

• p_from_mw: Active power flow into the line at “from” bus (MW)
• q_from_mvar: Reactive power flow into the line at “from” bus (MVAr)
• p_to_mw: Active power flow into the line at “to” bus (MW)
• q_to_mvar: Reactive power flow into the line at “to” bus (MVAr)

Table 5 shows an extract of the data file *CINELDI_MV_reference_grid_base_branch_extra.csv* that defines data on the branches in the reference system in addition to those branch parameters defined in the standard MATPOWER case format [8].

**Table 5:** Example of additional branch data from CINELDI_MV_reference_grid_base_branch_extra.csv.

type	length_km	installation_ year	location_type
TXSE 3 × 1 × 240 Al	0.64340	2004	semi-urban
TXSE 3 × 1 × 240 Al	0.39981	2004	semi-urban
TXSE 1 × 3 × 240 Al	0.44674	2011	semi-urban
…	…	…	…

The data columns in *CINELDI_MV_reference_grid_base_branch_extra.csv* (Table 5) are defined as follows:

• type: Line type according to the Norwegian type designation, with reference to Table 21 (see the *Experimental design, materials, and methods* section)
• length_km: Line length (kilometres)
• installation_year Year that the line was installed
• location_type Type of the location of the line (``urban'', ``semi-urban'' or ``rural'') according to the classification in [1]

In the base version of the grid presented, it is assumed that the location of all lines is classified as semi-urban.

Table 6 shows an extract of the data file *CINELDI_MV_reference_grid_base_bus_extra.csv* that define data for the load points (at a subset of the nodes) in the reference system in addition to those bus (node) parameters defined in the standard MATPOWER case format [8].

**Table 6:** Example of additional load point data from CINELDI_MV_reference_grid_base_bus_extra.csv.

bus_i	constant_impedance	constant_current	constant_power
2	0	0	1
6	0	0	1
8	0	0	1
…	…	…	…

The data columns in Table 6 specify the parameters of a ZIP load model, similarly as the input data format used for instance in pandapower [9]:

• bus_i: The bus (node) number, with reference to Table 3
• constant_impedance: The share of the load demand (the Pd and Qd columns in Table 3) associated with a constant impedance load [0,1]
• constant_current: The share of the load demand (the Pd and Qd columns in Table 3) associated with a constant current load [0,1]
• constant_power: The share of the load demand (the Pd and Qd columns in Table 3) associated with a constant power load [0,1]

The values of the three columns should add up to 1 for all load points. In the default data for the base version of the reference system, all loads are set to be constant power.

### 2.2 Technical and economic data for standard grid components

For distribution system planning studies considering grid investment alternatives, one needs additional technical and economic data for the grid components one considers installing. Tables 7 and 8 show technical data and cost data for a set of standard underground cable types (*standard_underground_cable_types.csv*) and overhead line types (*standard_overhead_line_types.csv*), respectively, for a 22 kV distribution grid.

**Table 7:** Technical and economic data for installation of new underground cables (standard_underground_cable_types.csv).

type	*R_ohm_per_km*	*X_ohm_per_km*	*Cd_nF_per_km*	*Imax_A*	cost_NOK_per_km_rural	cost_NOK_per_km_semi-urban	cost_NOK_per_km_urban)
TSLE 3 × 1 × 50 Al / 16	0.641	0.22	160	200	601 747	735 541	1 018 229
TSLE 3 × 1 × 95 Al / 25	0.320	0.20	200	295	640 884	774 678	1 057 366
TSLE 3 × 1 × 150 Al / 25	0.206	0.19	230	370	708 481	842 275	1 124 963
TSLE 3 × 1 × 240 Al / 35	0.125	0.17	280	465	761 603	895 397	1 178 085

**Table 8:** Technical and economic data for installation of new overhead lines (standard_overhead_line_types.csv).

type	R_ohm_per_km	X_ohm_per_km	Cd_nF_per_km	Imax_A	cost_NOK_per_km
40-AL1/7-ST1A (FeAl nr. 25 6/1)	0.723	0.394	9.226	266	605 173
80-AL1/13-ST1A (FeAl nr. 50 6/1)	0.360	0.373	9.793	416	697 375
111-AL1/19-ST1A (FeAl nr. 70 6/1)	0.258	0.362	10.100	517	759 408

The data columns in Tables 7 and 8 are defined as follows:

• type: Line type according to the Norwegian type designation
• R_ohm_per_km: Resistance in ohm per km
• X_ohm_per_km: Reactance in ohm per km
• Cd_nF_per_km: Mutual capacitance *C*_d_ of the lines in nF per km
• Imax_A: Current carrying capacity *I*_max_ in ampere represents the maximal allowed continuous current, or long-term thermal limit, of the lines
• Cost_NOK_per_km: Investment costs in NOK per km (as explained in detail below, Table 8 contains three cost columns for three location types)

The costs of distribution lines are investment costs including the cost of the actual grid components as well as costs of personnel, engineering and installing the components. The investment costs of underground cables also include the costs of preparing trenches for different types of locations, classified in [1] as urban, semi-urban and rural^6^. All costs are given in the Norwegian currency NOK for cost level 2021. The costs do not include earth conductors, downlead of cable from pylons, consideration of existing pipes and cables (for power lines or other infrastructures) or discarding costs or residual values of existing cables. For consistency with the recommended analysis horizon for socio-economic analyses of grid investment projects in Norway [10], the default value for the economic lifetime for distribution lines and distribution substations is assumed to be 40 years.

Table 9 shows technical data and cost data for a set of standard distribution substations in a 22 kV distribution grid (*standard_substation_types.csv*). Distribution substations transforming down to 230 V instead of 400 V may be more common in rural Norwegian distribution grids. Only substations with secondary voltage 400 V are included in Table 9 for *S*_N_ > 100 kVA, but the cost of corresponding 22 kV / 230 V substations can be estimated by using the cost difference between 230 V and 400 V for the 100 kVA substation in Table 9. As for distribution lines, the default value for the economic lifetime of distribution substations is assumed to be 40 years.

**Table 9:** Technical and economic data for installation of new substations (standard_substation_types.csv).

type	*SN_kVA*	P0_W	Pk_W	ek	cost_NOK
22 kV / 230 V (by H pylon)	100	240	1200	3.6	213 268
22 kV / 400 V (by H pylon)	100	240	1200	3.6	188 613
22 kV / 400 V	315	615	2900	4.3	288 784
22 kV / 400 V	500	880	3900	4.6	345 299
22 kV / 400 V	800	1220	6300	4.9	400 794
22 kV / 400 V (inside building)	800	1220	6300	4.9	568 103
22 kV / 400 V	1250	1960	9700	5.7	482 621
22 kV / 400 V	1600	2150	13,100	6.2	474 943

The data columns in Table 9 are defined as follows:

• type: Voltage level and type of distribution substation
• SN_kVA: Rated power *S*_N_ of transformer (kVA)
• P0_W: No-load loss *P*_0_ of transformer (W)
• Pk_W: Load loss *P*_k_ of transformer (W)
• ek: Total short-circuit voltage *e*_k_ of transformer (%)
• cost_NOK: Total investment costs (NOK)

## 3 Load Data

The load data associated with the reference system consist of a set of hourly load demand time series for a full year, the customer type composition of the load demand for each time series, a mapping between these time series and the nodes in the grid model, and a set of scenarios for the long-term (10-year) development of load demand in the system. These data describe an extended version of the reference system with information on possible new load points and flexibility resources being added to the system in the future.

Table 10 gives an overview of the files in the load data set.

**Table 10:** Overview of load data set.

File name	Description of data
load_data_CINELDI_MV_reference_system.csv	Set of 104 normalized hourly load demand time series
mapping_loads_to_CINELDI_MV_reference_grid.csv	Mapping of the load time series to nodes in the reference grid data set
share_load_per_customer_type.csv	Share of the accumulated load demand (energy consumption) that is comprised by different customer types for each of the load time series in the load data set
time_series_IDs_irregular_load.csv	List of load time series IDs for load time series that have been defined as irregular
time_series_IDs_primarily_residential.csv	List of load time series IDs for load time series that are primarily residential
scenario_LEC_only.csv	Scenario for long-term load development with only local energy communities (LECs) as new loads
scenario_LEC_fewer.csv	Scenario for long-term load development with new LECs but fewer than in scenario LEC_only.csv
scenario_LEC_even_fewer.csv	Scenario for long-term load development with new LECs but even fewer than in scenario LEC_fewer.csv
scenario_LEC_and_FCS.csv	Scenario for long-term load development with new LECs and two new FCSs
scenario_LEC_and_one_FCS.csv	Scenario for long-term load development with new LECs and one new fast-charging station (FCS)

### 3.1 Load time series

An extract of the load demand time series in *load_data_CINELDI_MV_reference_system.csv* is presented in Table 11. The first column is the time stamp on the format ``DD/MM/YY HH'', where hour 01 is the time interval 00:00–01:00, etc. The load data are based on the average load demand measured over each hour. The column heading of the other 104 columns is a load time series ID. Note that this means that there are more load time series in the load data set than there are load points in the grid data set. The reason is that the load data were collected for a larger grid area than the grid data set and then mapped to the grid data set as described below. Each time series corresponds to the aggregated load demand measures at a distribution substation. The load time series are normalized so that the maximum value for each time series is 1.0. A load time series for a given load point in absolute values (e.g., units kW) can be obtained by multiplying a normalized load time series with a load demand scaling factor (e.g., in units kW). This scaling factor will then be the maximum load demand value for the load point during the year that is considered.

**Table 11:** Extract of load data set with time series of normalized load demand per hour (load_data_CINELDI_MV_reference_grid.csv).

Time	1	2	3	…	104
01/01/18 01	0.352193	0.062411	0.444571	…	0.995012
01/01/18 02	0.576884	0.23078	0.559109	…	0.132861
…	…	…	…	…	…
31/12/18 24	0.413032	0.81016	0.895164		0.081373

Each load time series in the load data set represents one or several customer types (``agricultural'', ``residential'', etc.) .^7^ Each time series is a sum of time series for different customer types. The data are aggregated in this way to ensure anonymity, but the customer type composition for each load time series is indicated in the data file *share_load_per_customer_type.csv*, which is illustrated in Table 12. The values are the share of the annual energy consumption for each load point that is comprised by different customer types. Thus, summing the values for the different customer types gives the sum 1.0 for each row in Table 12.

**Table 12:** Extract of the file share_load_per_customer_type.csv defining the share of the accumulated load demand (energy consumption) of each load time series that is comprised by different customer types.

Time_series_ID	residential	agriculture	public	industry	commercial
1	0.9809	0.0011	0.0179	0.0000	0.0000
2	0.6959	0.3041	0.0000	0.0000	0.0000
3	0.9556	0.0131	0.0091	0.0223	0.0000
…	…	…	…	…	…
104	1.0000	0.0000	0.0000	0.0000	0.0000

### 3.2 Reference system extended with new loads

**Fig. 2:**
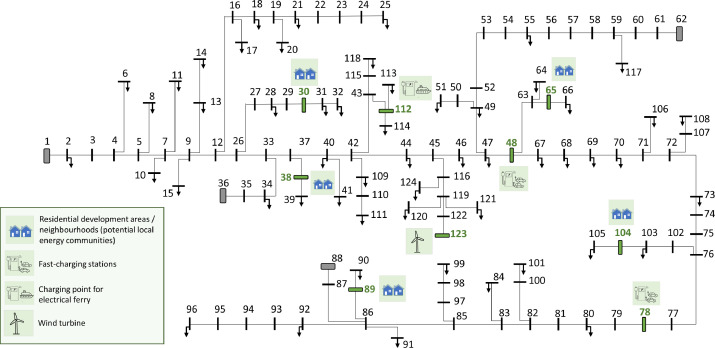
CINELDI MV reference system extended with new loads.

The base version of the reference system with grid data as described above only includes information on load points presently existing in the distribution system. The load data part of the reference data set also defines an extended version of the reference system with information on possible new load points and potential flexibility resources [11] being added to the system in the future. Table 13 and Fig. 2 gives an overview of the potential new loads in this extended version of the reference system compared to the base reference system. All these load points are located at nodes that have no load points in the base reference system. The new load points have been placed so that they can cause grid challenges in different parts of the grid and so that they can be supplied through different backup feeders after grid reconfiguration.

**Table 13:** New loads that can be added to the reference system.

Node IDs	Type	Description
30, 38, 65, 89, 104	Residential development areas (neighbourhoods, potential LECs)	Typically, 0.58–0.88 MW peak load. Power factor 0.95 lagging by default. A basic, aggregated representation of these loads are described in the following subsections. (More detailed representations can be constructed by bottom-up modelling of load/generation components such as residential loads, PV, local energy storage (batteries), and EV charging.)
48, 78	Fast-charging stations (FCS)	Centralized, high-power electric vehicle charging stations. Typically, 12–16 charging points with 125 kW capacity, corresponding to an aggregated converter rating of 1.5–2.0 MVA in total for all charging points. Power factor 1.0 by default (but can be assumed to be controllable).
112	Ferry charging point	High-power charging point. 1 unit with capacity 1–4 MW (10 min charging time). Power factor 1.0 by default (but can be assumed to be controllable).
123	Wind turbine	Wind turbine (or small distributed wind power plant) with unspecified capacity, connected to the grid using a power electronics converter. (Further details are not specified in this data set.)

### 3.3 Mapping of load time series to the reference grid

To include the load time series in the CINELDI reference system, a mapping is needed that specifies which of the 104 load time series of the load data set is to be used for which of the 124 nodes of the reference grid. This mapping is given in the file *mapping_loads_to_CINELDI_MV_reference_grid.csv*. The structure of this is shown in Table 14, where bus_i refers to the grid data set (cf. Table 3) and time_series_ID refers to the load data set. The value in the column existing_load is ``True'' for load points existing in the base system and ``False'' for new load points that can be included as part of scenarios for the future load development in the system. There are 54 existing load points (i.e., nodes with nonzero load) in the base system. In addition, new (potential future) load points are indicated in Fig. 2, including five local energy communities (LECs). These new loads can be included as part of scenarios for the future load development in the system. The objective of the mapping in Table 14 is to assign load time series to each of these 54+5 = 59 nodes in the reference system.

**Table 14:** Mapping between the load time series and nodes in the reference grid (mapping_loads_to_CINELDI_MV_reference_grid.csv).

bus_i	time_series_ID	existing_load
2	1	True
6	2	True
8	3	True
…	…	…
30	20	False
38	24	False

**Fig. 3:**
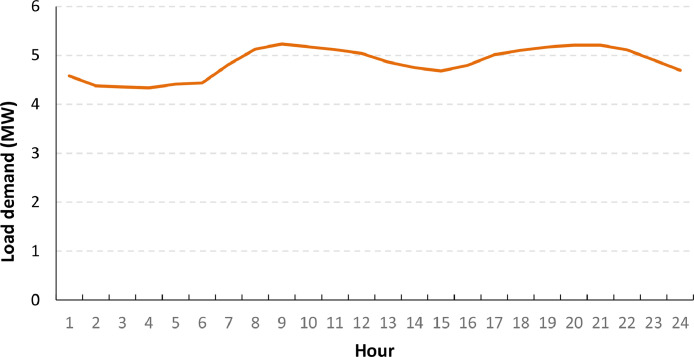
Total load demand on February 28 for the base version of the CINELDI MV reference system.

When the load time series are mapped to the load points of the base system (not including any new loads), the annual maximum load demand for the system is 5.231 MW. Note that this peak load value is lower than the sum of the peak load of the individual load points (6.407 MW, cf. Table 2) because their annual maximums do not occur simultaneously. The annual maximum for the system occurs on February 28, and Fig. 3 shows the time variation of the load demand in the system this day.

### 3.4 Scenarios for long-term load development

**Fig. 4:**
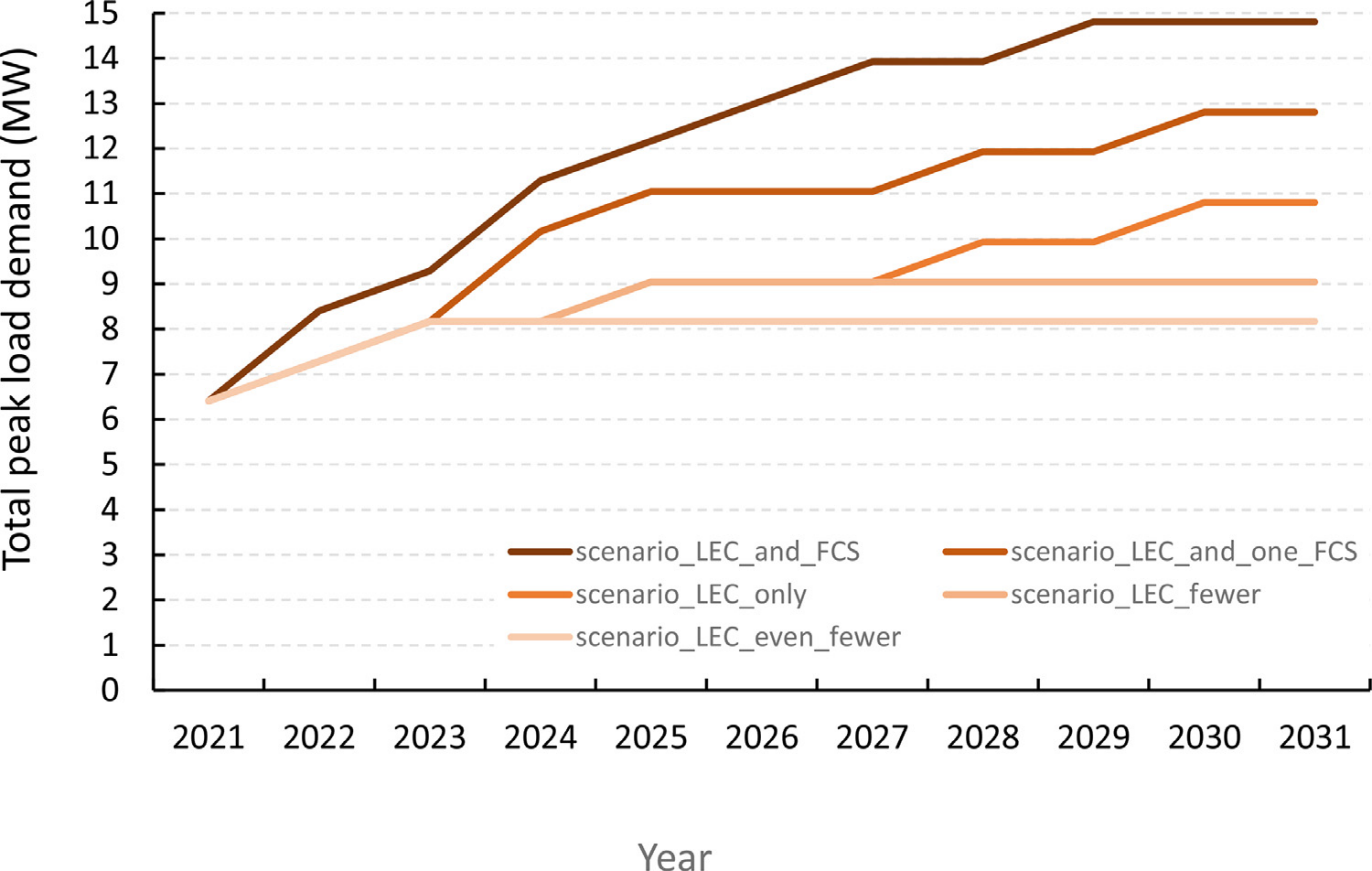
Scenarios for representing the uncertainty in long-term load demand forecasts. In the scenario labels, LEC is an abbreviation of Local Energy Community and FCS is an abbreviation of Fast-Charging Station.

Fig. 4 illustrates three scenarios for the long-term load development over a 10-year planning horizon. The reference year (year 0) is set to be 2021. For simplicity, it is assumed that the only change from year to year is that new point loads are added to the system. A description of the scenarios and reference to the data file defining them is given in Table 15.

**Table 15:** Load development scenarios shown in Fig. 4.

Label (data reference)	Scenario description
scenario_LEC_only	Gradual load growth, with new residential development areas (neighbourhoods that are potential LECs) being added to the system throughout the planning horizon
scenario_LEC_fewer	Similar to scenario_LEC_only, but where the load growth only persists until 2025 and no new development areas (neighbourhoods) are adding load to the grid after that
scenario_LEC_even_fewer	Similar to scenario_LEC_fewer, but where only the two neighbourhoods already under development until 2023 are included, but future development plans are not realized
scenario_LEC_and_one_FCS	Similar to scenario_LEC_only, but in addition a fast-charging station is added in 2024
scenario_LEC_and_FCS	Scenario with large-scale electrification of transportation, with two fast-charging stations (FCS) added to the system

As an example, data for scenario_LEC_and_FCS is specified in Table 16, and the scenario data are specified by the following fields:

• year_rel: Year relative to the reference year (2021)
• bus_i: Bus (node) number in the reference grid (cf. Table 3)
• load_added_MW: Peak load in MW added to the new load point at the node
• label: The type and/or identifier of the load^8^
• power_factor: Power factor (lagging) (optional column)

**Table 16:** Example definition of load development scenarios including load points (scenario_LEC_and_FCS.csv).

year_rel	bus_i	load_added_MW	label	power_factor
1	78	2.00	FCS_highway	1.00
2	89	0.88	LEC	0.95
3	48	2.00	FCS_shopping_mall	1.00
4	104	0.88	LEC	0.95
5	65	0.88	LEC	0.95
6	30	0.88	LEC	0.95
8	38	0.88	LEC	0.95

New load points are added to nodes without load points in the base grid data set. Unless the optional power_factor column is added, the reactive power is given by an identical and constant power factor 0.95 lagging. The new load points representing potential local energy communities (label ``LEC'') have a primarily residential load composition. For FCSs, the amount of added load refers to the aggregated converter rating, with a power factor that in principle is controllable.

Fig. 4 shows the resulting total peak load in the system for the scenarios, summing over the peak load of the individual load points.

Note that the default assumption for the load scenarios is that the same normalized load time series (based on measurements made in 2018) are applicable for all years of the planning horizon. Such a simple baseline assumption is necessary in absence of more sophisticated modelling and prediction of future load behaviour, but the reader should be aware that the time dependence and variability in the future and in the past will in general not be the same.

## 4 Reliability Data

The reliability data associated with the reference system consist of the reliability data (failure frequencies and outage times) of lines and information on switchgear located at the ends of some of these lines. An overview of the files is given in Table 17.

**Table 17:** Overview of reliability data set.

File name	Description of data
CINELDI_MV_reference_system_switchgear.csv	Data on the location and position of switchgear (disconnectors and circuit breakers) in the reference grid
CINELDI_MV_reference_system_reldata.csv	Reliability data for lines in the reference grid
CINELDI_MV_reference_system_load_point.csv	Load point data for reliability of supply calculations
reldata_for_component_types.csv	Reliability statistics for main types of distribution grid components

**Fig. 5:**
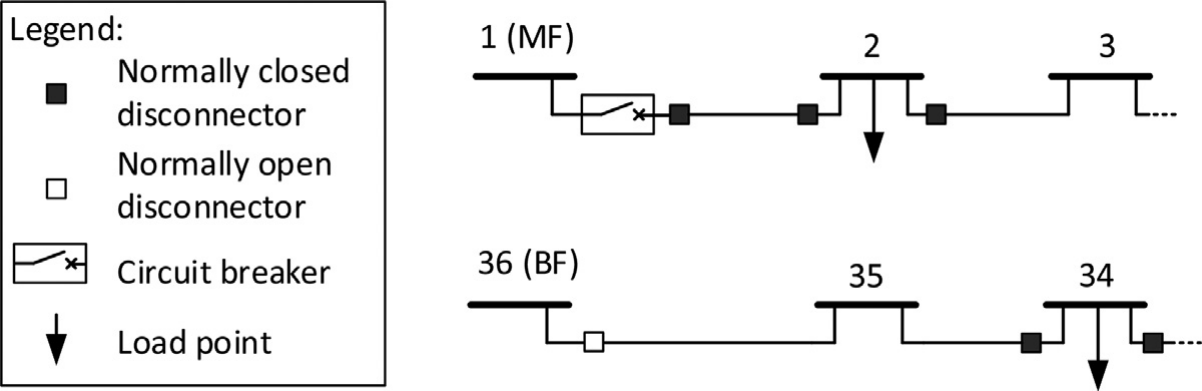
Switchgear information for the reference system, including circuit breakers and disconnectors.

Fig. 5 illustrates the information included about the switchgear in the reference system. Disconnectors (switches) are located by all the nodes with load points that represent the distribution substations in the grid. All these disconnectors are normally closed (NC) but can be opened to section the grid in case of faults. Circuit breakers (CBs) are located by the main feeder (MF) and the backup feeders (BF). The disconnectors by the backup feeders are normally open but can be closed in fault situations to reconfigure the grid to restore power supply to the parts of the grid that are isolated after sectioning to isolate the fault^9^. Switchgear data are given in the file *CINELDI_MV_reference_system_switchgear.csv*, and an extract illustrating the data format is given in Table 18.

**Table 18:** Example switchgear data from CINELDI_MV_reference_system_switchgear.csv.

f_bus	t_bus	circuit_breaker	disconnector_closed
1	2	True	True
1	2	False	True
2	1	False	True
2	3	False	True
..	…	…	…

The data columns in *CINELDI_MV_reference_system_switchgear.csv* (Table 18) are defined as follows:

• f_bus: Bus (node) number for the node by which the switchgear is situated (``from bus'', cf. Table 3)
• t_bus: Bus (node) number for the node at the other end of the line connected to the switchgear (``to bus'', cf. Table 3)
• circuit_breaker: ``True'' if the switchgear is a circuit breaker, ``False'' if it is a disconnector
• disconnector_closed: ``True'' if disconnector is closed, ``False'' if disconnector is open

The f_bus and t_bus columns refer to the bus (node) numbers defined in the MATPOWER-format bus matrix (cf. Table 3). Note that each line (cf. Table 4) may have one or more switchgear components at either end. Since the f_bus and t_bus numbers in Table 18 are used to define *which* end the switchgear are connected to, the t_bus and f_bus numbers may be interchanged in Table 18 compared to the corresponding line entry in Table 4.

The data columns in *CINELDI_MV_reference_system_reldata.csv* (Table 19) are defined as follows:

• f_bus: ``from bus'' number (cf. Table 3)
• t_bus: ``to bus'' number (cf. Table 3)
• lambda_perm: Failure frequency for permanent faults [12] (per year)
• lambda_temp: Failure frequency for temporary faults [12] (per year)
• r_perm: Outage time (including repair time) for permanent faults (hours)
• r_temp: Outage time for temporary faults (hours)
• sectioning_time: Sectioning time after failure (hours)

**Table 19:** Example distribution line reliability data from CINELDI_MV_reference_system_reldata.csv.

f_bus	t_bus	lambda_perm	lambda_temp	r_perm	r_temp	sectioning_time
1	2	0.011517	0.001094	5	0.02	0.5
2	3	0.007157	0.00068	5	0.02	0.5
3	4	0.013179	0.001252	5	0.02	0.5
…	…	…	…	…	…	…

Information on the customer types of the end-users comprising the load points is relevant for calculating customer interruption costs. Sufficient data for a simplified calculation of interruption costs (or costs of energy not supplied) are presented below. More detailed calculations can be carried out by using the load data presented in the previous subsection and information on the Norwegian cost of energy not supplied (CENS) scheme [13,14]. How such interruption cost calculations can be carried out will not be explained in detail in this data article^10^.

Simplified load point data sufficient for interruption cost calculation are illustrated in Table 20. Here, it is assumed only one of the customer types (``agricultural'', ``residential'', etc.^7^) according to the Norwegian CENS scheme are present at each of the load points. It is also assumed that expected annual interruption costs for a load point will be calculated by multiplying the annual expected energy not supplied (ENS) measured in kWh for the load point by some specific interruption cost $c_{\text{ENS}}$ in units NOK/kWh (cost level 2021). The value of $c_{\text{ENS}}$ will in practice vary with the time of occurrence and duration of the power supply interruption, but in Table 20, $c_{\text{ENS}}$ values are only stated as average values for two typical interruption durations.

**Table 20:** Example load point data for the reliability of supply calculations from CINELDI_MV_reference_system_load_point.csv.

bus_i	customer_type	c_NOK_per_kWh_1 h	c_NOK_per_kWh_4h
2	residential	60.32	52.74
6	residential	52.74	46.12
8	residential	60.21	52.65
…	…	…	…
124	residential	54.62	47.76

The data columns in *CINELDI_MV_reference_system_reldata.csv* (Table 20) are defined as follows:

• bus_i: Bus (node) number in the reference grid (cf. Table 3)
• customer_type: Customer type according to the Norwegian cost of energy supplied (CENS) scheme [13]
• c_NOK_per_kWh_1h: Average specific interruption cost for a power supply interruption with duration 1 hour (NOK/kWh)
• c_NOK_per_kWh_4h: Average specific interruption cost for a power supply interruption with duration 4 hours (NOK/kWh)

## 5 Experimental Design, Materials and Methods

### 5.1 Grid data

The grid data are based on data for a real Norwegian medium voltage (22 kV) distribution system. The original data set was acquired from the Network Information System (NIS) of a grid company. These data were first provided for research purposes under a non-disclosure agreement in a previous project before being adapted to serve as a reference grid in the research centre CINELDI.

In Norway, grid data are generally defined as power system sensitive information and is subject to confidentiality. Therefore, the original data set had to be anonymized and was moreover processed to obtain a simplified but still representative reference data set for a Norwegian distribution system.

The original data set described a meshed distribution grid operated radially. Only one main feeder (radial) has been included for further processing from the grid described in the original data set. Only the grid's topology and impedances, in addition to the components’ MVA rating, were included in the processed data set. That is to say: No geographic coordinates are included in the grid data set, all numbering/identification from the original data set has been removed and replaced with a new consecutive numbering, and other information on other components, such as transformers and switches, has been removed.

In the processed grid data set, 59 nodes have a potential load point associated with them. For each of these load points, one value for the load (active power) is defined. These load values ​​represent the maximum power consumption at the load point during a typical year. There is no one-to-one correspondence between these load values ​​in the grid data set and the load values ​​in the load data set; the load values ​​in the grid data set are based on received data but scaled and aggregated so that it cannot be directly traced back to the original data set.

In the grid data set, the 54 load points represent existing loads in the grid are based on aggregation and redistribution of the substations in the original grid data set. The 5 remaining of the 59 load points mentioned above represent potential future loads.

Information on the line type, length, and installation year of the individual lines was not included in the original data set. Values for these parameters were therefore reconstructed by comparing their impedances and ratings to a table with data for line types present in the original grid data^11^. This table can be found in the file *distribution_line_types_in_reference_grid.csv*, and an extract is given in Table 21. Component installation year was collected from the original data set for the components where it was available. In some cases where component installation year was not available, the year of the last time the component was modified was used. This estimate gives an upper bound on the actual installation year. In case neither installation year nor modification time was available in the original data set, it was assumed that lines of the same type have the same installation year.

**Table 21:** Extract of overview of line types present in the original grid data set from distribution_line_types_in_reference_grid.csv.

type	main_type	R_ohm_per_km	X_ohm_per_km	Cd_nF_per_km	Imax_A
BLX 50	Overhead line (1–22 kV)	0.36	0.297471	11.74588	245
Cu 16	Overhead line (1–22 kV)	1.127	0.333341	10.4795	115
FeAl 25	Overhead line (1–22 kV)	0.724	0.333699	10.46825	235
…	…	…	…	…	…

The data columns in *distribution_line_types_in_reference_grid.csv* (Table 21) are defined as follows:

• type: Line type according to the Norwegian type designation
• main_type: Description of the main type of component that the line belongs to, either overhead line or underground cable, including voltage level
• R_ohm_per_km: Resistance (ohm/km)
• X_ohm_per_km: Reactance (ohm/km)
• Cd_nF_per_km: Mutual capacitance (nF/km)
• Imax_A: Thermal (long term) rating (ampere)

Lengths for the lines are calculated by dividing the line resistance in the grid data by the unit length resistance of the corresponding standard cable type. Line charging susceptances had to be estimated according to the π-model for lines and MATPOWER data format [8] using the capacitance values in Table 21 and the estimated branch length. The code for processing the grid data is available through GitHub [16] as the script *process_grid_data.py*.

Technical and economic data for the set of standard grid components are extracted from the Norwegian handbook for grid planning [1]. The included component types are selected since they are expected to be likely choices for new installations in Norwegian MV distribution grids. For underground cables, the cable types are extracted from the table in [1] for 24 kV TXSE/TSLE/TSLF single-line cables laid flatly in the trench and covered^12^. For overhead lines, the line types are extracted from the table in [1] for 24 kV overhead lines suspended in a plane with 1.5 m distance between the phases^13^.

### 5.2 Load data

The original load data set obtained from the distribution grid company contained load demand values (in kW measured as an average per hour) per customer type per distribution substation. These load data are not temperature corrected. To anonymize the data, the substation numbering from the original load data set has been removed and replaced with new consecutive numbering (from 1 to 104). Furthermore, load demand values for the different customer types were aggregated to one time series per distribution substation. The share of total energy consumption over the year per customer type is calculated for each substation and stated separately (Table 12).

The aggregated time series for each distribution substation is divided by the maximum consumption value (in kW) for the substation. The processed time series thus contains a relative consumption time series for the year (relative to maximum consumption that year) with values ​​in the interval [0,1].

To ensure anonymity, information on the maximum energy consumption value for the substations is not preserved in the processed load data set. It is therefore not possible to identify with certainty the number of end-users that form the basis of a load time series.

The grid data set and the load data set were initially processed independently, and load data were collected for a larger grid area than the grid data set. There is no one-to-one correspondence between the 104 load time series and the 59 load points in the processed grid and load data sets. To use these data sets together, 59 of the load time series therefore had to be selected and assigned to a load point (and thus a node) in the grid data set. This mapping is described below and implemented in the script *create_load_mapping.py* available through GitHub [16].

The new local energy community load points represent residential development areas with a high degree of flexibility. To establish a baseline scenario without flexibility, they are modelled using residential area load time series from the load data sets. Next, the 54 existing load points were assigned load time series. Time series ID 1 is assigned to the first node with a load point, time series ID 2 is assigned to the second node with a load point, and so on, excluding those time series that already have been assigned to LEC nodes or that had been defined as irregular^14^.

The processing and mapping of grid and load data ensures that they cannot be used to identify individual end-users: The location of the load time series in the grid topology is arbitrary, and there is no connection between the real location of the substations in the real grid and this new location of the load time series in the grid topology of the processed grid data set.

The information in Table 13 about potential new loads due to electrification of transportation (fast-charging of electric vehicles and electric ferries) are based on [17].

### 5.3 Reliability data

The set of reliability data parameters included for the reference system is based on the necessary input data for the RELRAD methodology for reliability analysis of radial distribution grids [18]. Moreover the data format is based on the RelDist.jl implementation of RELRAD [19] that is publicly available through GitHub^15^.

Failure rates are based on statistics from the Norwegian standardised system FASIT for collection, calculation and reporting of disturbance and reliability data [20]. Table 22 shows average failure rates for distribution system component derived from the FASIT system, including data for all of Norway for the years 2012–2021. The raw disturbance and outage data underlying these failure rates are confidential. The data in Table 22 are combined with the line lengths to produce the line reliability data shown in Table 19. The code for preparing the line reliability data is available through GitHub [16] as the script *prepare_reldata.py*.

**Table 22:** Average reliability data for distribution system components from Norwegian fault statistics (reldata_for_component_types.csv).

main_type	lambda_perm	lambda_temp	r_perm	r_temp
Overhead line (1–22 kV)	3.97	4.34	3	0.02
Underground cable (1–22 kV)	1.79	0.17	5	0.02
Distribution transformers	0.54	0.16	4	0.02
Circuit breakers (1–22 kV)	0.14	0.29	n/a	n/a
Disconnectors (1–22 kV)	0.12	0.04	n/a	n/a

The repair times of components registered in the FASIT system are associated with large uncertainties, as described in detail in [20]. Therefore, the outage times in Table 22 were estimated by the authors as typical values for Norwegian MV distribution grids based on the experience from several previous projects and analyses. For instance, the outage time values for permanent faults used in [19] were set to 4 h for both overhead lines and underground cables. The analysis of repair times in FASIT data presented in [20], estimated mean values of 3.2 h, 4.7 h and 3.6 h for overhead lines, underground cables and distribution transformers, respectively. Compared to the values in [20], the values for distribution lines in Table 22 were for the purposes of this paper rounded to the closest integer number of hours because of the large uncertainty in the data basis. Doing so, they are rounded down for overhead lines and up for underground cables, which also reflect the expectation that repair times of underground cables typically are longer. The outage times for temporary faults are based on the values used in [19], which in turn are based on expert judgement by a local distribution grid company.

The data columns in *reldata_for_component_types.csv* (Table 22) are defined as follows:

• main_type: Description of the main type of component that the line belongs to, either overhead line or underground cable, including voltage level
• lambda_perm: Failure frequency for permanent faults (per 100 km per year, or per 100 components per year)
• lambda_temp: Failure frequency for temporary faults (per 100 km per year, or per 100 components per year)
• r_perm: Expected outage time for permanent faults (hours per failure)
• r_temp: Expected outage time for temporary faults (hours per failure)

Typical values for specific interruption costs $c_{\text{ENS}}$ in units NOK/kWh were stated in Table 20 for each load point. These values are the customer interruption costs for a power supply interruption of a certain duration r but with the time of occurrence averaged over the year.

The specific interruption costs $c_{\text{ENS}}$ as a function of r were calculated according to the formula [21]

$$c_{\text{ENS}}\left(r\right)=\frac{c_{\text{ref}}\left(r\right)}{r}\cdot f_{c}\cdot \frac{P_{\text{ref}}}{P_{\text{avg}}}.$$

The specific interruption cost functions $c_{\text{ref}}\left(r\right)$ and the values of the correction factors $f_{c}$ are based on the Norwegian CENS scheme [13]. $P_{\text{ref}}$ is the load demand for the reference time and $P_{\text{avg}}$ is the average load demand over the year. The specific interruption cost parameter $c_{\text{ENS}}$ is calculated for all five customer types in the reference system using the parameter values stated Table 23.

**Table 23:** Data for customer interruption cost calculation for the customer types in the reference system.

customer_type	ratio_P_ref_P_avg	f_c	c_ref_1h	c_ref_4h
residential	2.6	0.96	25.86	90.46
agriculture	2.5	0.88	23.55	81.66
public	2.3	0.38	214.04	317.93
industry	3.0	0.38	145.92	451.52
commercial	2.5	0.49	242.43	580.17

The columns in Table 23 are defined as follows:

• ratio_P_ref_P_avg: Ratio $P_{\text{ref}}/P_{\text{avg}}$ between the load demand for the reference time $P_{\text{ref}}$ and the average load demand over the year $P_{\text{avg}}$
• f_c: Average correction factor $f_{c}\;$for customer interruption costs over the year when using interruption cost functions in units NOK/kW
• c_ref_1h: Specific interruption cost function $c_{\text{ref}}\left(r\right)$ at the reference time evaluated for an interruption duration r= 1 hour
• c_ref_4h: Specific interruption cost function $c_{\text{ref}}\left(r\right)$ at the reference time evaluated for an interruption duration r= 4 hours

Typical values for $P_{\text{ref}}/P_{\text{avg}}$ in Table 23 were estimated for each customer type by calculating the average annual load demand relative to the annual peak load demand for each load point in the reference system classified as that customer type. Correction factors $f_{c}$ were taken from [14]. Cost functions with cost level 2017 in [13] were corrected for the increase in consumer price index from 2017 to 2021. The code for calculating the values in Table 23 is available through GitHub [16] as the script *prepare_reldata.py*.

## Ethics Statements

The raw load and grid data have been anonymized so that it is impossible to identify individual end-users or grid components based on the processed load and grid data sets.

## CRediT authorship contribution statement

**Iver Bakken Sperstad:** Data curation, Formal analysis, Investigation, Methodology, Software, Visualization, Writing – original draft. **Olav Bjarte Fosso:** Conceptualization, Data curation, Formal analysis, Investigation, Methodology, Software, Supervision, Validation, Visualization, Writing – review & editing. **Sigurd Hofsmo Jakobsen:** Formal analysis, Methodology, Software, Writing – review & editing. **Arnt Ove Eggen:** Data curation, Resources, Validation, Writing – review & editing. **Julie Helen Evenstuen:** Data curation, Investigation, Software, Visualization, Writing – original draft. **Gerd Kjølle:** Conceptualization, Funding acquisition, Project administration, Supervision, Validation, Writing – review & editing.

## Declaration of Competing Interest

The authors declare that they have no known competing financial interests or personal relationships that could have appeared to influence the work reported in this paper.

## Data Availability

Reference data set for a Norwegian medium voltage power distribution system (Original data) (Zenodo).

## Footnotes

3 In this article, the term ``node'' is used for what is commonly referred to as ``bus'' in power grid models because some of the nodes in the grid model do not represent physical busbars. Still, the term ``bus'' is used in connection with data formats where the term is used.

4 In this article, the term ``distribution line'' or ``line'' is used for what is commonly referred to as ``branch'' in power grid models. Still, the term ``branch'' is used in connection with data formats where the term is used.

5 The available power capacity of the main feeder (node 1) may be lower than the power transfer capacity of the downstream lines due to the power rating of the HV/MV transformer or by the power capacity of the upstream HV grid. The power capacity available at the backup feeder may be reduced due to insufficient reserve capacity in the neighbouring distribution systems. The system can be modified by defining backup feeders to be connected to a) other HV/MV substations or b) another backup feeder in the same reference system (giving the grid a ring topology).

6 In Norwegian, the classification is ``byområde'', ``forstad'' og ``landsbygd''. This simplified classification does not consider local factors such as for instance the amount of mountain and rock along the route or the need for archaeological surveys. Actual installation costs may therefore have large variations between projects for the same location type with the same cable lengths.

7 These customer types are used in the Norwegian Cost of Energy Not Supplied scheme as described in the sections on the reliability part of the data set. The corresponding Norwegian terms for the customer types are, in the order listed in Table 12: ``husholdning'', ``jordbruk'', ``offentlig virksomhet'', ``industri'', ``handel og tjenester''. In addition, the customer type ``industri med eldrevne prosesser'' (which can be translated to ``energy-intensive industries'' in English) is defined in Norwegian regulation, but this customer type is not represented in the data set.

9 It is assumed that there are disconnectors that can isolate the distribution transformer and the LV grid in case of faults on the distribution transformer or in the LV grid. But since there are no distribution transformers or LV grids included in the data set, such disconnectors are not included in the data set either.

10 Strictly speaking, it requires more detailed data than provided here to calculate end-user interruption costs according to the CENS scheme and the requirements for reporting of disturbance data (FASIT) in Norway [15]: This requires interruption costs to be calculated for each individual end-user based on the time of occurrence and duration of each power supply interruption, and it requires information on the time variation of load demand throughout the year for the customer type the end-user belongs to. However, this data is provided for the purpose of more aggregated reliability of supply analysis where one assumes that information is not available about the time dependence of individual customer types represented by a load point.

11 The original grid data includes a type of ``fictitious'' line segments that represent parts of nodes in substations, connections between line segments, etc. These appear as nearly-zero-impedance lines with very small lengths. The processed data set includes 27 such lines.

12 Norwegian title of table in [1] for the types of underground cables: ``24 kV TXSE/TSLE/TSLF (enleder i jord, flat forlegning)''.

13 Norwegian title of table in [1] for the types of overhead lines: ``24 kV luftledning (planoppheng med jordline, E-mast, 1,5 m faseavstand)''.

14 For this work, a qualitative assessment has been made of which load time series are ``irregular'' in the sense of deviating from common and clearly recognizable seasonal, weekly, and diurnal patterns. There are multiple reasons why real load time series may be irregular: It may be due to measurement errors, measurements missing in parts of the period (e.g., when new loads are connected to the grid), changes of industrial processes during the year, or simply due to the particularities of the load behaviour of individual end-users.

15 https://github.com/SINTEF-Power-system-asset-management/RelDist.jl

## Footnotes

1 CINELDI (Centre for Intelligent Electricity Distribution) is a Norwegian Centre for Environmental-friendly Energy Research (FME), 2016–2024, www.cineldi.no.

2 In this article, the term ``investment costs'' is consistently used to describe the cost of installing new grid components and not the historical costs of installing the components already existing in the grid. When existing components are replaced by new components, the term ``reinvestment cost'' would be more precise.

8 For FCSs, the label is used to refer to the location and identifier of the charging station load time series in a separate data set under preparation.
